# Impact of GDMT on outcomes after mitral valve edge-to-edge repair stratified by SMR proportionality

**DOI:** 10.1007/s00392-025-02599-3

**Published:** 2025-02-05

**Authors:** Lukas Stolz, Daniel Kalbacher, Benedikt Koell, Nicole Karam, Tania Puscas, Marco Metra, Marianna Adamo, Maximilian Spieker, Patrick Horn, Holger Thiele, Tobias Kister, Ralph-Stephan von Bardeleben, Philipp Lurz, Karl-Patrik Kresoja, Christos Iliadis, Roman Pfister, Mohammad Kassar, Fabien Praz, Bruno Melica, Teresa Trenkwalder, Erion Xhepa, Michael Neuss, Christian Butter, Paul Grayburn, Jörg Hausleiter

**Affiliations:** 1 Medizinische Klinik Und Poliklinik I, Klinikum Der Universität München, Marchioninistr. 15, 81377 Munich, Germany; 2 German Center for Cardiovascular Research (DZHK), Partner Site Munich Heart Alliance, Munich, Germany; 3 Department of Cardiology, University Heart and Vascular Centre Hamburg, Hamburg, Germany; 4 German Center of Cardiovascular Research (DZHK), Partner Site, Hamburg/Kiel/Lübeck, Germany; 5 Department of Cardiology, European Hospital Georges Pompidou, and Paris Cardiovascular Research Center (INSERM U970), Paris, France; 6 Cardiac Catheterization Laboratory and Cardiology, ASST Spedali Civili and University of Brescia, Brescia, Italy; 7 Heart Center, Department of Cardiology, University Hospital of Düsseldorf, Düsseldorf, Germany; 8 Department of Cardiology, Heart Center Leipzig at University of Leipzig, Leipzig, Germany; 9 Zentrum Für Kardiologie, Johannes-Gutenberg-Universität, Mainz, Germany; 10 Department III of Internal Medicine, Heart Center, University of Cologne, Cologne, Germany; 11 Universitätsklinik Für Kardiologie, Inselspital Bern, Bern, Switzerland; 12 Centro Hospitalar Vila Nova de Gaia, Espinho, Portugal; 13 Deutsches Herzzentrum München, Technische Universität München, Munich, Germany; 14 Herzzentrum Brandenburg, Medizinische Hochschule Brandenburg Theodor Fontane, Bernau, Germany; 15 Division of Cardiology, Department of Internal Medicine, The Heart Hospital, Baylor Scott & White, Dallas, TX USA

**Keywords:** GDMT, Heart failure, Secondary mitral regurgitation, Proportionality

**Fig. 1:**
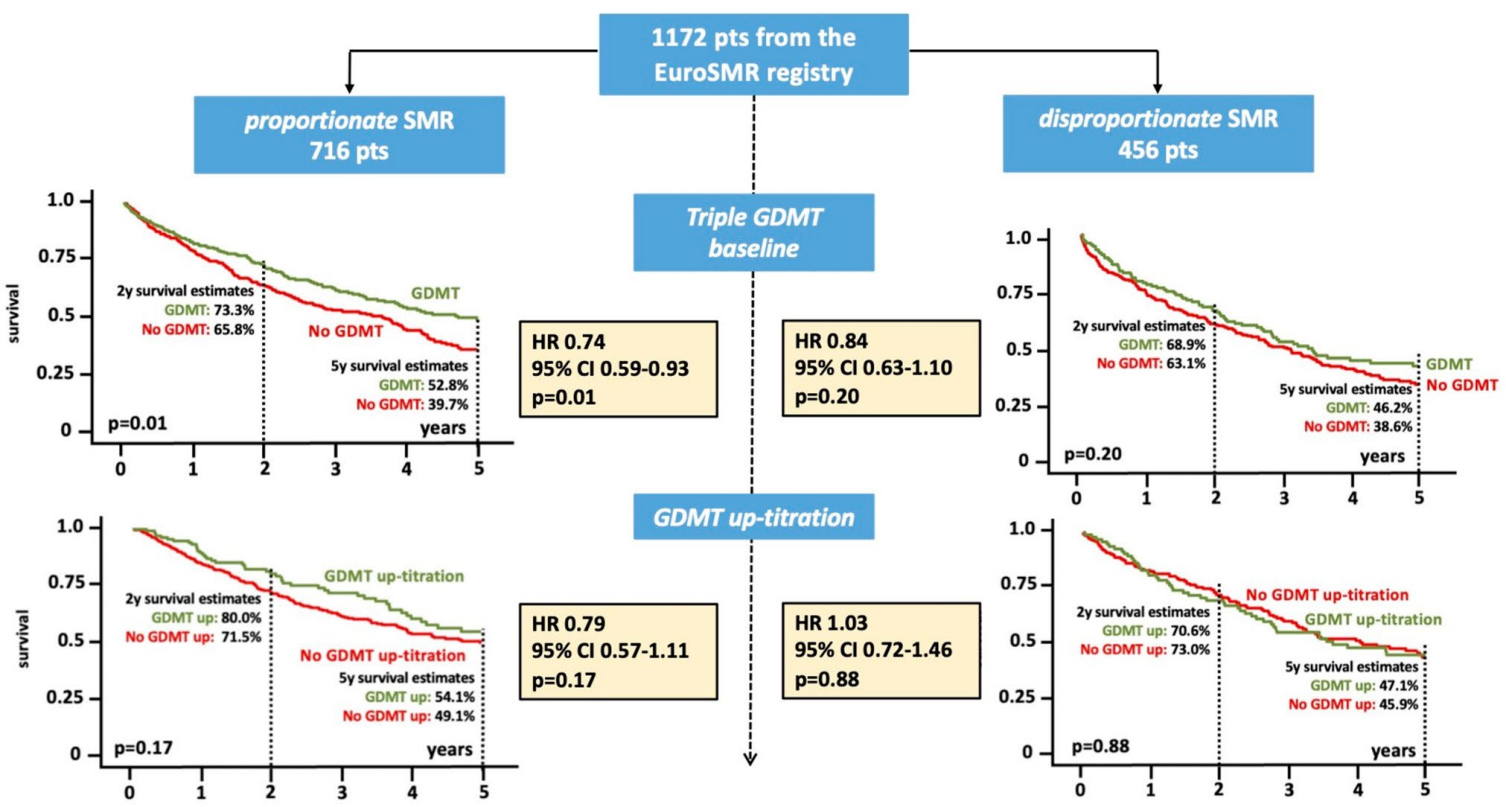
Impact of GDMT in M-TEER patients stratified by SMR proportionality. Application and up-titration of triple GDMT were associated with significantly higher 5-year survival rates in patients with proportionate SMR. Survival rates in patients with disproportionate SMR did not differ depending on GDMT application. *GDMT* guideline directed medical therapy, *M-TEER* mitral valve transcatheter edge-to-edge repair, *SMR* secondary mitral regurgitation

Sirs:

Transcatheter mitral valve edge-to-edge repair (M-TEER) has emerged as a guideline-recommended procedure for the treatment of secondary mitral regurgitation (SMR). In 2018, two large randomized-controlled trials presented diverging results regarding the benefit of M-TEER beyond guideline directed medical therapy (GDMT). The most popular explanation for these contradictory study results is the concept of “MR proportionality” [1]. This pathophysiological framework relates MR severity (effective regurgitant orifice area [EROA]) to the dilatation of the left ventricle [left-ventricular end diastolic volume (LVEDV)]. SMR is referred to as proportionate if MR severity is explainable by the extend of LV dilation. In those patients, GDMT application is believed to be effective and may lead to LV reverse remodeling with subsequent reduction of SMR. In patients with disproportionate MR, quantitative SMR parameters exceed the degree of LV dilation mostly due to concomitant pathological processes. In patients with disproportionate MR, the application of GDMT is hypothesized to have less effect on SMR severity, which necessitates further interventional treatment (e.g., M-TEER) to reduce or eliminate SMR [2]. The aim of this study was to investigate the impact of GDMT application on symptomatic outcomes and long-term survival in patients undergoing M-TEER for severe SMR stratified by SMR proportionality (Fig. 1).

The study included patients from the EuroSMR registry (European Registry of Transcatheter Repair for Secondary Mitral Regurgitation) with left-ventricular ejection fraction (LVEF) ≤ 50% and available information on SMR proportionality and GDMT. Using the mean EROA/LVEDV ratio as cut-off (0.00179 cm^2^/ml), patients were stratified into proportionate (low EROA/LVEDV ratio) and disproportionate MR (high EROA/LVEDV ratio). Triple GDMT was defined as simultaneous application of a beta-blocker (BB), renin–angiotensin system inhibitor (RAS-I) and a mineralocorticoid receptor antagonist (MRA). We then investigated the impact of baseline (prior to M-TEER) GDMT application and GDMT up-titration during follow-up on 5-year survival. The study was approved by each center local ethics committee and adheres to the principles outlined in the declaration of Helsinki.

The study included 1172 patients (31.3% women, mean age 72.6 ± 10.2 years). MR was severe (4 +) in 43.0%, moderate-to-severe (3 +) in 52.3%, and moderate (2 +) in 4.7% of patients. Mean MR EROA and RegVol were 0.30 ± 0.15 cm^2^ and 42.6 ± 20.1 ml, respectively. LVEF was reduced (31.4 ± 9.3%), while right-ventricular function was borderline [Tricuspid annular plain systolic excursion (TAPSE) 16.5 ± 4.7 mm]. 456 patients (38.9%) were classified as disproportionate SMR and 716 patients (61.1%) as proportionate SMR. The percentage of patients with triple GDMT was 32.7% in patients with disproportionate and 39.1% in those with proportionate SMR (*p* = 0.026).

Application of triple GDMT was associated with significantly higher 5-year survival rates in patients with proportionate SMR (GDMT 52.1% vs. no GDMT 39.7%, *p* = 0.010). The predictive value of triple GDMT in the setting of proportionate SMR was confirmed in a multivariate Cox regression model (hazard ratio 0.745; confidence interval 0.588–0.942, *p* = 0.014). In contrast, for patients displaying disproportionate SMR, although there was a trend, the utilization of GDMT did not result in statistically significant differences in 5-year survival (GDMT 46.2% vs. no GDMT 38.6%, *p* = 0.204). GDMT up-titration after M-TEER was observed in 28.5% of patients with proportionate SMR and in 32.6% of patients with disproportionate SMR (*p* = 0.233). Among patients with proportionate SMR, the up-titration of GDMT after M-TEER was associated with a numeric difference toward better 5-year survival rates (GDMT up-titration 54.1% vs. no GDMT up-titration 49.1%, *p* = 0.168). Conversely, no discernible effect was observed in patients with disproportionate SMR (*p* = 0.878).

In the present study, we were able to demonstrate that GDMT application and further up-titration after M-TEER are important survival predictors in patients with proportionate SMR. To date, the effect of GDMT application according to proportionality in M-TEER patients has not been investigated [3, 4]. Our findings support the hypothesis that GDMT is of particular significance in patients with homogenous and proportionate SMR. Within the context of the proportionality concept, it is currently believed that the favorable response to pharmacological heart failure GDMT may explain the neutral results observed in the MITRA-FR study, as patients with proportionate MR were more likely to benefit from it. However, in patients with disproportionate MR, heterogeneous comorbidities which lead to inhomogeneous remodeling (asymmetric tethering, etc.) seem to reduce the effectiveness of GDMT. This may be reflected in the reduced prognostic value of pharmacological treatment in patients with disproportionate MR within the EuroSMR registry, with a growing belief that M-TEER might be of particular importance in those patients. This hypothesis is supported by the positive results of the COAPT trial.

Right-ventricular dysfunction was not an exclusion criterion in the MITRA-FR study as it was in COAPT and might have contributed to heart failure symptoms and mortality in those patients. Even though not being investigated so far, GDMT might also have an impact on RVD, potentially providing an additional explanation to the difference in results between the two studies. The present study is subject to several limitations. Besides absence of core laboratory supervision, patients were mainly treated at a time when SGLT-2 inhibitors were not yet recommended by heart failure guidelines. Although this is one of the largest databases of patients treated with M-TEER, the numbers may have been relatively small for subgroup analysis.

In accordance with the “SMR Proportionality concept,” the application and further up-titration of heart failure GDMT appears to be of particular prognostic importance in patients with “proportionate SMR”.
